# Evolutionary Factor Analysis

**DOI:** 10.6028/jres.093.035

**Published:** 1988-06-01

**Authors:** K. Schostack, P. Parekh, S. Patel, E. R. Malinowski

**Affiliations:** Department of Chemistry and Chemical Engineering, Stevens Institute of Technology, Castle Point, Hoboken, NJ 07030

Because of chemical interconversion, many chemical systems cannot be physically separated, making chemical identification and quantification difficult. The spectra (IR, UV, Visible, Raman, CD, etc.) of such systems exhibit overlapping contributions of uncataloged components, confounding the identification as well as the quantification. Strategies based on factor analysis [1], a chemometric technique for handling complex multi-dimensional problems, are ideally suited to such problems. Abstract factor analysis (AFA) reveals the number of spectroscopically visible components. Evolutionary factor analysis (EFA) [2–4] takes advantage of experimental variables that control the evolution of components, revealing not only the concentration profiles of the components but also their spectra even when there are no unique concentrations or spectral regions.

Evolutionary factor analysis makes use of the fact that each species has a single, unique maximum in its evolutionary concentration distribution curve. We have recently applied this self-modeling method to the infrared spectra of stearyl alcohol in carbon tetrachloride solution. The evolutionary process of this system was achieved by increasing the concentration of stearyl alcohol from 0.0090 to 0.0800 g/L in 15 stages, each time recording the IR spectra from 3206 to 3826 cm^−1^. The spectra were corrected for baseline shift, solvent absorption and reflectance losses. The 15 spectra were then digitized every 3 cm^−1^ and assembled into a 35 × 15 absorbance matrix [*A*] appropriate for factor analysis.

The factor indicator function [1], the reduced eigenvalue [5] and cross validation [6] indicated that three species contribute to the observed spectra. Thus AFA expresses the data matrix as a product of a 35×3 absorptivity matrix [*E*]_abst_ and 3×15 abstract concentration matrix [*C*]_abst_.

$$[A]={\left[E\right]}_{\text{abst}}\;{\left[C\right]}_{\text{abst}}$$

Because the abstract matrices are mathematical solutions devoid of chemical meaning, they must be transformed into physically meaningful absorptivities and concentrations. This is accomplished by target transformation factor analysis (TFA) [1], a powerful technique which allows one to test factors *individually* without requiring any *a priori* information concerning the other factors. A test vector *C*_test_ emulating an evolutionary profile can be converted into a predicted vector *C*_pred_ that lies completely inside the factor space by finding a transformation vector *T* that minimizes the sum of squares of the difference between *C*_test_ and *C*_pred_. Accordingly, *C*_pred_ is given by:

$$C_{\text{pred}}=T{\left[C\right]}_{\text{abst}}$$

in which *T*=*C*_test_ [*C*]′_abst_ *C*]_abst_ [*C*]′_abst_}^‒1^

The prime indicates matrix transposition.

These equations were used to target test 15 ideal (Dirac delta function) concentration profiles, represented by uniqueness tests for each column of the absorbance matrix. A uniqueness vector consists of zeros for all elements except the one in question, which contains unity:

$$\begin{array}{ccc} C_{1} & = & \left(1,0,0,\ldots ,0,0,0\right)\\ C_{2} & = & \left(0,1,0,\ldots ,0,0,0\right)\\ \cdot & \cdot & \cdot \\ \cdot & \cdot & \cdot \\ C_{15} & = & \left(0,0,0,\ldots ,0,0,1\right) \end{array}$$

The three predicted vectors with maxima corresponding to the unique point of the respective test vector were retained as likely candidates. These crude profiles were refined, individually, by applying simplex optimization to the respective transformation vector, using a response function designed to minimize negative concentration points and double maxima in the profile.

Further refinement was achieved by the following iteration. Because negative regions are meaningless, all data beyond the boundaries marked by the first negative regions encountered on the left and on the right of the peak maximum were truncated. These profiles were normalized so the sum of squares equals unity and then assembled into a concentration matrix [*C*]. The pseudoinverse equation

$$\left[E]=[A][C{]}^{\prime }\;\{[C][C{]}^{\prime }\right\}$$

was used to calculate the spectral matrix, followed by another pseudoinverse

$$\left[C]={\left\{[E{]}^{\prime }\;[E]\right\}}^{-1}[E][A\right]$$

to recalculate the concentration profiles. This process (truncation, normalization and pseudoinverse followed by pseudoinverse) was repeated until no further refinement occurred.

The concentration profiles and spectra of the three unknown components of stearyl alcohol in carbon tetrachloride obtained in this manner were found to make chemical sense.

This EFA procedure, unlike others, was successful in extracting concentration profiles from situations where one component profile was completely encompassed underneath another component profile.

## References

[1] Malinowski ER, Howery DG (1980). Factor Analysis in Chemistry.

[2] Gemperline PG (1984). J Chem Inf Comput Sci.

[3] Vandeginste BGM, Derks W, Kateman G (1985). Anal Chim Acta.

[4] Gampp H, Maeder M, Meyer CJ, Zuberbuhler AD (1985). Talanta.

[5] Malinowski ER (1987). J Chemometrics.

[6] Wold S (1978). Technometrics.

